# Restraint Induces Sickness Responses Independent of Injection with Epstein-Barr Virus (EBV)-Encoded dUTPase

**DOI:** 10.4236/jbbs.2014.411049

**Published:** 2014-11-15

**Authors:** Taryn G. Aubrecht, Bachir Abi Salloum, Maria Eugenia Ariza, Marshall Williams, Brenda Reader, Ronald Glaser, John Sheridan, Randy J. Nelson

**Affiliations:** 1 Departments of Neuroscience, The Ohio State University, Columbus, OH, USA; 2 Departments of Molecular Virology, Immunology & Medical Genetics, The Ohio State University, Columbus, OH, USA; 3 Institute for Behavioral Medicine Research, Wexner Medical Center, Comprehensive Cancer Center, The Ohio State University, Columbus, OH, USA; 4 Division of Bioscience College of Dentistry, The Ohio State University, Columbus, OH, USA

**Keywords:** Epstein-Barr Virus, Restraint Stress, Sickness Response, Anxiety-Like and Depressive-Like Behaviors

## Abstract

Most adult humans have been infected by Epstein-Barr virus (EBV), a putative cause of chronic fatigue syndrome, and carry latent EBV. The EBV-encoded dUTPase can induce sickness responses in mice and chronic stress exacerbates this response. Because individuals often adapt to chronic stress, we tested the hypothesis that acute restraint stress would potentiate these sickness responses elicited by EBV-encoded dUTPase. Male CD-1 mice were injected daily for one or three days with either saline or EBV-encoded dUTPase. Additionally, mice from each condition were either restrained for three hours daily or left undisturbed during the light phase when mice are inactive. Restraint decreased weight gain during the one- and three-day experiments. Restraint in saline injected mice increased anxiety-like behavior in the open field during the three-day experiment. There were no behavioral differences during the one-day experiment. Restraint stress had no effect when experienced acutely on one day, but did produce a sickness response after three days of exposure regardless of saline or dUTPase injection. In contrast to the effects of chronic stress and EBV-encoded dUTPase on the sickness response, acute stress did not affect sickness responses in association with EBV-encoded dUTPase. Thus, dUTPase does not appear to provoke the same sickness responses after acute stress as compared to chronic stress.

## 1. Introduction

Chronic fatigue syndrome (CFS) is characterized by severe fatigue, myalgia, lymphadenopathy, sore throat, stress, and depression [1]. This constellation of symptoms is termed “sickness response”, and is conserved across many mammalian species and functions to aid in recovery from illness [2]. Many symptoms of CFS can be exacerbated by stress.

Stress can modulate the immune system [3]; acute stress typically enhances, whereas chronic stress tends to impair immune function [4]. Conversely, an exaggerated stress response leads to the prolonged production of glucocorticoids that bind glucocorticoid receptors within immune cells and alter their function [5]. Furthermore, exposure to stress alone induces sickness behavior [6]. Stress, independent of immune challenge, can elevate glucocorticoids and modulate cytokine signaling to influence immune function [7]. Physical and psychological (restraint) stress in rats increases plasma interleukin-6 (IL-6), a proinflammatory cytokine associated with fever [8] [9]. Adrenalectomy increases fever following lipopolysaccharide (LPS) and stress; although, adrenalectomy with corticosterone replacement to mimic levels observed in stressed rats, reduced fever compared to adrenalectomy alone. Treatment of adrenal intact rats with a glucocorticoid receptor antagonist increases plasma levels of IL-6 and fever compared to vehicle treated animals [10]. These data suggest that glucocorticoids are involved in inhibitory feedback to control the immune response but also contribute to the development of sickness behavior.

CFS is thought to be caused or triggered by Epstein-Barr virus (EBV) in the subset of patients who develop CFS following acute mononucleosis [1]. One of the EBV-encoded proteins, deoxyuridine triphosphate nucleotidohydrolase (dUTPase), is capable of inducing immune dysregulation in vitro by inhibiting proliferation of peripheral blood mononuclear cells and altering production of proinflammatory cytokines [11]-[14]. EBV-encoded dUTPase induces sickness responses in mice, characterized by increased body temperature and reduced locomotor activity similar to the sickness response observed following LPS challenge [15]-[17]. Daily injections for two weeks with 10 or 20 μg of EBV-encoded dUTPase increased body temperature and reduced locomotor activity compared to saline-injected mice [15] [18]. With the exception of some members of the genera *Mollicutes* [19], all free-living organisms, as well as some viruses, encode for a dUTPase (EC 3.6.1.23) [20].

dUTPases represent a family of metalloenzymes that catalyze the hydrolysis of dUTP to dUMP and pyrophosphate [20], thus preventing dUTP from being incorporated into DNA by DNA polymerases. dUTPases can be divided into at least three subgroups based upon their structure and specificity for dUTP. The largest subgroup, which exhibits a high specificity for dUTP, is the homotrimeric dUTPases, which are found in plants, animals, fungi, bacteria, and some viruses, including the retroviruses and poxviruses. These homotrimeric dUTPases are composed of three identical polypeptides aligned so that three highly conserved amino acids domains (domains 1, 2 and 4) on one polypeptide interact with the amino acids in conserved domain 3 on an adjacent peptide. The catalytic site is completed with the binding of dUTP to the flexible C-terminal end of the third polypeptide, which contains the conserved amino acids in domain 5. Thus, all three polypeptide subunits contribute to the formation of a catalytic site and there are three catalytic sites per holoenzyme. The homotrimeric dUTPase is essential for the replication of *E. coli* [21] and *Saccharomyces cerevisiae* [22], and probably for human cells because no mutants have been isolated deficient in this activity. The monomeric dUTPases, which are thought to have arisen from the trimeric dUTPases by gene duplication [23], are found exclusively in herpesviruses [24]. Structural data of the EBV-encoded dUTPase has demonstrated that the single catalytic site of the enzyme is comprised of the five highly conserved domains characteristic of the homotrimeric and monomeric dUTPase; thus, the catalytic site of EBV-encoded mimics the catalytic site of the homotrimeric dUTPases [25]. Sequencing analyses have recently demonstrated that the herpesviruses’ dUTPases contain an additional conserved domain (domain 6) that is absent in the homotrimeric dUTPases [26]. It has been suggested that this novel herpesvirus-specific domain may contribute to some novel function, but that function(s) has not been elucidated. The last group, the homodimeric dUTPases, identified in *Leshmania major* [27] [28], *Trypansoma cruzi* [29], *Caenorhabditis elegans* [30], and *Campylobacter jejuni* [31] differ not only structurally from the monomeric and trimeric dUTPases, but they also exhibit broader substrate specificity. Furthermore, sequence comparisons have demonstrated that the dimeric dUTPases lack the five consensus amino acid motifs found in mono and trimeric dUTPases and that they are evolutionary related to the dCTPase-dUTPase of bacteriophages T2 and T4 [28]. EBV-encoded dUTPase induces sickness responses in mice [18]. However, the acute effects of EBV-encoded dUTPase remain unspecified, particularly in conjunction with a psychological stressor.

Other components of the sickness response, such as anxiety- and depressive-like behavior, observed following LPS or proinflammatory cytokine administration are unexplored with EBV-encoded dUTPase injections [16] [17]. Increased depressive-like behavior during sickness is likely caused, in part, by prolonged inflammation [17] [32]. Elevated inflammation is a known component of the pathophysiology of EBV infection [33]. Therefore, injections of dUTPase from EBV might produce similar alterations in anxiety- and depressive-like behaviors. LPS and IL-1*β* both induce sickness responses and increase anxiety-like behavior [34]. Thus, we examined anxiety- and depressive-like responses after EBV-encoded dUTPase injections in the present study.

To determine the short-term duration of EBV-encoded dUTPase injections required to elicit a change in anxiety- and depressive-like behaviors and the sickness response, as well as how a psychological stressor alters the sickness response, mice were injected with EBV-encoded dUTPase for one or three days and restrained for 3 h each day. Following the final injection, anxiety- and depressive-like behaviors were assayed. Given the ability of stress to alter immune function, we hypothesized that restraint would exacerbate sickness behavior elicited by EBV-encoded dUTPase.

## 2. Materials and Methods

### 2.1. Subcloning and Purification of EBV-Encoded dUTPase

The subcloning of the EBV (BLLF3 pET3A kindly provided by Dr. Peter Sommer (Institut fur Mikrobiologic und Hygiene, Abteilung Virolgie)) was conducted by PCR amplification using the forward (5’-CCGGTTAAGCTTGGATCCATGGAGGCCTGTC-3’ and reverse (5’-GCGAATTCTCATTGACCCGACGATCC-3’) primer sets (125 pmol of each), DNA (140 ng), high fidelity PCRsupermix (Invitrogen, Gary Island, NY, USA) and the following PCR conditions: Denaturation at 94°C for 3’ (1 cycle) followed by 35 cycles of 94°C for 30 seconds (sec), 50°C for 30 sec, 72°C for 1’ and one cycle at 72°C for 20’. The PCR product was purified using the QIA quick gel extraction kit (QIAGEN) and cloned into the protein expression vector pTrcHis Topo (Invitrogen, Gary Island, NY, USA). Twenty individual clones were isolated following transformation of *E. coli* Top 10 competent cells, DNA was then purified using the QIA Prep Spin Miniprep kit (QIAGEN, Valencia, CA, USA), screened by PCR for the presence of specific dUTPase genes and the sequence verified by DNA sequencing analysis. The pTrcHisdUT constructs, containing the EBV-encoded dUTPase gene in the correct orientation and in frame, were used to transform *E*. *coli* BL21(DE3)*plyS* competent cells for purification of recombinant proteins as described below.

The recombinant EBV-encoded dUTPase protein was purified using HisPur^™^ Spin columns (3 ml resin bed) as described by the manufacturer (Pierce, Rockford, IL, USA). Briefly, BL21(DE3)*plyS* containing a specific PTrcHisDUT construct was grown in LB medium containing chloramphenicol (25 μg/ml) and ampicillin (100 μg/ml) at 37°C for 2.5 h IPTG (1 mM final concentration) was added and the culture was incubated an additional 2 h at 37°C. Bacteria were collected from 36 liters of medium by low speed centrifugation and the bacterial pellet was resuspended in 50 ml of extraction buffer (50 mM sodium phosphate, 300 mM NaCl and 10 mM imidazole, pH 7.4). Bacteria were lysed by ultrasonication. The resulting homogenate was centrifuged (15,000 × g, 30 min at 4°C), and the supernatant was applied to a HisPur^™^ spin column, which was equilibrated in extraction buffer. The column was washed three times with two-resin bed volumes of extraction buffer and the EBV-encoded dUTPase protein was eluted by washing the column four times with one resin-bed volume of 50 mM sodium phosphate, 300 mM NaCl and 150 mM imidazole, pH 7.4. Fractions were assayed for the EBV-encoded dUTPase activity as described previously [11] and for protein using the Coomassie Brilliant Blue dye-binding assay (Bio-Rad Laboratories, Hercules, CA, USA) using bovine serum albumin as the standard. A unit of dUTPase activity was defined as the amount of enzyme required to convert 1 nM of dUTP to dUMP and pyrophosphate per min at 37°C under the assay conditions. Purity of EBV-encoded dUTPase was determined by SDS-PAGE as described previously [11]. Proteins were visualized using EZBlue^™^ protein gel stain as described by the manufacturer (Sigma Aldrich, St. Louis, MO). The EBV-encoded dUTPase protein preparations were tested as described previously [11] and were free of detectable levels of LPS, peptidoglycan (SLP-HS), DNA, or RNA. Purification of EBVdUTPase has previously been described; additionally, enzymatic activity is not necessary for EBVdUTPase to induce immune dysregulation *in vitro* [11] [15].

### 2.2. Animals

Six to eight-week old male CD-1 mice were obtained from Charles River, Inc. (Wilmington, MA, USA). Mice were group housed (n = 5/cage) in propylene cages (dimensions: 30 × 15 × 14 cm) at an ambient temperature of 22°C ± 2°C, relative humidity of 50% ± 10%, provided with food (Harlan Teklad 8640, Indianapolis, IN, USA) and filtered tap water ad libitum. Animals were allowed to acclimate to the facility and a 12:12 light/dark cycle (lights on: 6:00–18:00) for one week before the start of the experiment. Forty mice were used for the three-day injection experiment (n = 10/group) and 39 mice were used for the one-day injection experiment (n = 10/group except the restrained saline group, n = 9). The experiment was conducted in two sets, approximately 5 mice/group in each set.

### 2.3. Treatment with EBV-Encoded dUTPase and Restraint

Mice were injected intramuscularly into the right hindlimb daily, for one or three days, with either saline or 10 μg of EBV encoded-dUTPase suspended in saline between 8:30–9:00 h. Starting the first day of injections, half of the saline- and EBV-injected groups were restrained daily in ventilated 50 mL conical restraint tubes (3 h/day) at random times during the light phase. Thirty minutes of restraint stress is sufficient to induce increases in murine corticosterone [35].

### 2.4. Body Measurements

Body temperature was measured with a rectal thermometer (Harvard Apparatus, Holliston, MA, USA) twice daily, at 8:30 h (time of injections) and 17:30 h (just before lights off). Mice were weighed at the initiation and conclusion (day 2 or 4) of the study.

### 2.5. Open Field

On the final day of injections (day 1 or 3) mice were tested starting at the onset of the dark cycle for activity and anxiety-like behavior using the open field test. The open field test in mice characterizes exploration of a novel environment and indexes overall locomotor activity. Central tendency is the primary measure for anxiety-like behaviors, defined as the proportion of time spent in the center of the open field. Mice were allowed to acclimate to the room for 20 min before testing. Mice were placed in a 40 cm × 40 cm clear acrylic chamber. The center was defined as the central 28 cm × 28 cm. At the end of testing mice were placed in a clean cage. Mice were recorded for 10 min during the open field and tapes were assessed by a condition blind observer using observer software (Noldus Corp. Leesburg, VA, USA) for time in the center versus periphery and total moves during the test.

### 2.6. Forced Swim Test

Following the open field test the one day injection mice were assessed for depressive-like behaviors on the forced swim test as described previously [36]. We added the forced swim test as a second measure of depressive-like behavior following unexpected results in the forced swim test with the three-day injection experiment. Mice were placed in a 4000 mL glass beaker filled with 3000 mL of room temperature (22°C ± 2°C) water for five minutes. The video was scored on Observer software (Noldus Corp. Leesburg, VA, USA) by a condition-blind observer for total time floating, swimming, and climbing.

### 2.7. Tail Suspension Test

Following the open field or forced swim test, mice were tested for depressive-like responses using the tail suspension test, where more immobility equates to increased depressive-like behavior [37]. Mice were suspended by the distal part of their tail ~92 cm above the ground with electrical tape. Mice were recorded for 10 min and tapes were assessed by a condition-blind observer using Observer software (Noldus Corp. Leesburg, VA, USA) for time immobile and active.

### 2.8. Tissue Collection

Mice were placed in CO_2_ until nonresponsive, then their cervical spine was dislocated. Spleens were collected; in the 3-day experiment spleens were used for CD11b^+^ cell isolation. Because mice were injected in the right hind limb, inguinal and popliteal lymph nodes were removed only from the right side of each mouse.

### 2.9. RNA Isolation and Real-Time PCR from Pooled Lymph Nodes and Spleen (1-Day Restraint)

Pooled popliteal and inguinal lymph nodes, as well as whole spleen, were harvested for separate data analyses using quantitative PCR. RNA was collected from homogenized, pooled popliteal and inguinal lymph nodes and spleen from each of the animals in the control and experimental groups using TRIzol reagent/isopropanol precipitation (catalog #15596–026, Life Technologies, Grand Island, NY, USA). Concentrations of RNA were determined using spectrophotometry (Eppendorf, Hauppauge, NY, USA) and RNA was reverse transcribed to cDNA using the High Capacity cDNA Reverse Transcription kit (catalog #4368814, Life Technologies, Grand Island, NY, USA). Quantitative PCR was performed using the Applied Biosystems Assay-on-Demand Gene Expression protocol. In brief, experimental cDNA was amplified by real-time PCR where a target cDNA (e.g., IL-1*β*, IL-6, TNF (tumor necrosis factor), and (chemokine (C-C motif) ligand 2) CCL2) and a reference cDNA (glyceraldehyde-3-phosphate dehydrogenase (GAPDH)) were amplified simultaneously using an oligonucleotide probe with a 5’ fluorescent reporter dye (6-FAM) (20XTaqman Gene Expression Assays from Life Technologies, Grand Island, NY, USA). Fluorescence was determined on an ABI PRISM 7000-sequence detection system (Life Technologies, Grand Island, NY, USA) for 40 cycles. Data were analyzed with comparative threshold cycle (Ct) method and results are expressed as fold difference from GAPDH. All samples were run in duplicates, and data were analyzed using the 2^−ΔΔCt^ method with normalization to GAPDH with results expressed as fold change over the control group.

### 2.10. Cd11b^+^ Isolation and Nuclear Factor Kappa B (NFκB) Activation Assay (3-Day Restraint)

Spleens were harvested from mice and homogenized with ice-cold Hanks’ Balanced Salt Solution (Life Technologies, Grand Island, NY, USA) and mechanically disrupted using a Seward Stomacher 80 Biomaster system (Seward Laboratory Systems, Inc., Davie, FL, USA) to obtain single-cell suspensions as previously described in [38]. Briefly, cells were pelleted by centrifugation (1800 rpm for 10 min), and red blood cells were lysed with an ammonium chloride solution (0.16 M NH_4_Cl, 10 mM KHCO_3_, 0.13 mM EDTA). Cells were then resuspended in 10% FBS/HBBS, passed through a 70 μm filter (Fisher Scientific, Pittsburgh, PA, USA), pelleted by centrifugation, and resuspended in magnetic-activated cell sorting buffer (MACs) (PBS, (pH 7.2), 0.5% bovine serum albumin (BSA) and 2 mM EDTA). CD11b^+^ splenocytes were enriched by magnetic separation using CD11b microbeads as per manufacturer’s protocol (catalog #130-049-601; Miltenyi Biotec, San Diego, CA, USA). After separation, nuclear extract was isolated from CD11b^+^ cells using Nuclear Extract Kit (catalog #40010, Active Motif, Carlsbad, CA, USA) and protein quantification of nuclear extract was calculated using Bradford Assay (catalog #500–0205, BIO-RAD, Hercules, CA, USA) as per manufacturers’ protocols. Nuclear extract was lysed and activated transcription factor *NFĸB* was determined between experiment groups using the TransAMp65 *NFĸB* Activation Assay (catalog #40096, Active Motif, Carlsbad, CA, USA) as per manufacturer’s protocol. Groups were compared to vehicle control.

### 2.11. RNA Isolation and Real-Time PCR Array (3-Day Restraint)

RNA was collected from homogenized, pooled popliteal and inguinal lymph nodes from all the mice in each group using TRIzol reagent/isopropanol precipitation (catalog #15596–026, Life Technologies, Grand Island, NY, USA). RNA was then purified via DNase digestion and spin column clean-up using RNeasy Mini Kit (catalog #74104, QIAGEN Inc, Valencia, CA, USA). Concentration of RNA was determined using spectrophotometry (Denville, South Plainfield, NJ, USA) and RNA was reverse transcribed to cDNA using the RT^2^ First Strand Kit (catalog #330401, QIAGEN, Valencia, CA, USA). Quantitative PCR was performed using the RT^2^ Profiler PCR Arrays for Inflammatory Cytokines, Toll-like receptors, and NFκB Signaling Pathways (catalog #PAMM-011ZA, PAMM-018A, PAMM-025ZA, QIAGEN Inc, Valencia, CA, USA). In brief, experimental cDNA was amplified on an ABI PRISM 7300 sequence detection system (Life Technologies, Grand Island, NY, USA) by real-time PCR and normalized based on multiple reference cDNAs (GAPDH, beta-glucuronidase (GUSB), hypoxanthine-guanine phosphoribosyltransferase (HPRT), heat shock protein 90 kDa alpha class B member 1 (HSP90AB1), beta-actin (ACTB)). Data were analyzed with comparative threshold cycle method. Briefly, a fold change was calculated between two group (vehicle unrestrained, vehicle restrained, EBV unrestrained, EBV restrained) multiple comparisons were made to determine fold changes among all the groups, data are presented in graphs with vehicle unrestrained mice as the control sample. Because all individual samples from mice in each group were pooled there were no duplicates and therefore no error, so fold change was used to determine biological relevance.

### 2.12. Statistics

Main effects of restraint (no restraint versus restraint) and injection type (saline, EBV-encoded dUTPase) and interactions between the two variables were assessed. Changes in body temperature were assessed with a 2 × 2 multivariate ANOVA. Change in body mass, open field and tail suspension test were conducted as 2 × 2 univariate ANOVAs. Fold changes for IL-6, IL-1*β*, TNF, and CCL2 in the spleen and lymph nodes for the 1-day experiment were assessed with 2 × 2 univariate ANOVAs. Statistics were performed using SPSS 19 for Windows (IBM, Armonk, New York, USA). Mean differences were considered statistically significant when *p* was <0.05, outliers determined by Z score (±2 SD) were removed as specified in the results section. Significant differences were followed up with least significant differences post hoc tests. Because all the samples in the 3-day experiment were pooled to run the PCR array a fold change of 2.4 or greater was used to determine biological relevance [39].

## 3. Results

### 3.1. One Day of Treatment

#### 3.1.1. Body Temperature

**Figure 1.**
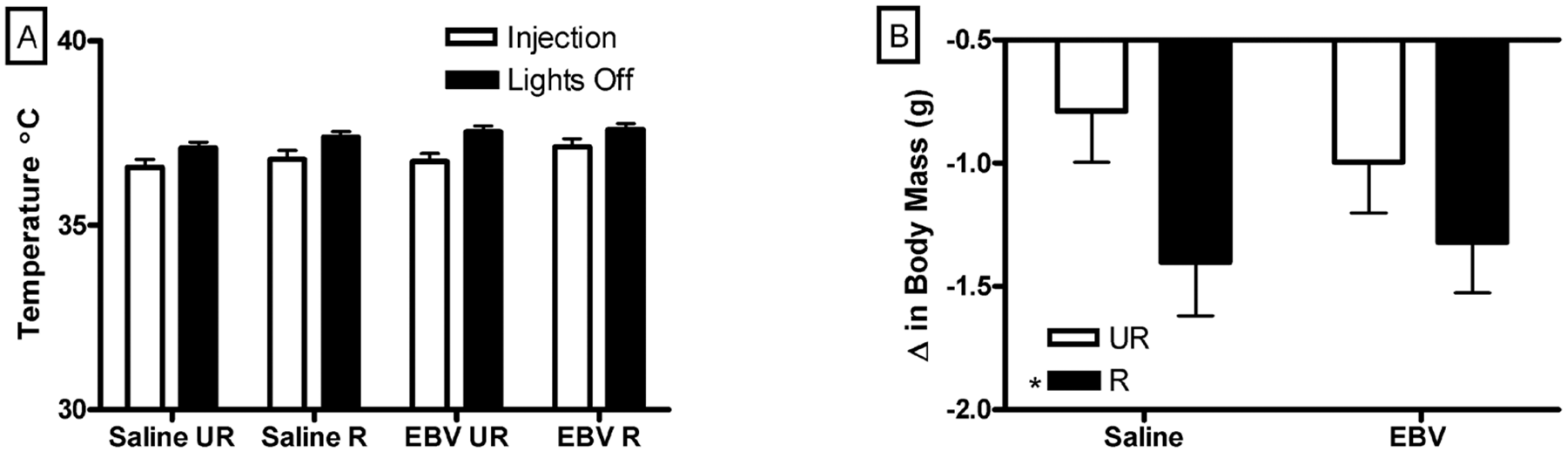
In the one day experiment, neither restraint nor EBVdUTPase injections affected body temperature. Temperature (±SEM °C) after injections (8:30) and just before lights out (17:30) on day one of injections, no significant differences in body temperature were observed (A); Restrained (R) mice lost more weight (±SEM) than unrestrained (UR) mice (B). Significant mean differences *p* < 0.05 indicated by asterisk (*) (n = 10/group except the restrained saline group, n = 9).

Body temperature was similar between saline and EBV-encoded dUTPase injected mice at both time points on day 1 (*p* > 0.05, Figure 1(A)). Restrained mice had a similar temperature to unrestrained mice on day 1 (*p* > 0.05, Figure 1(A)).

#### 3.1.2. Body Mass

Weight loss did not differ between saline or EBV-encoded dUTPase injected mice (*p* > 0.05). Restrained mice lost more weight than unrestrained mice (F_1,35_ = 5.06, *p* < 0.05, Figure 1(B)). Saline injected and restrained mice lost more weight than saline injected and unrestrained mice (*p* < 0.05, Figure 1(B)). Similarly there were no post hoc difference in weight loss between EBVdUTPase injected unrestrained and restrained mice (*p* > 0.05) or between saline and dUPTase injected and restrained mice (*p* > 0.05).

#### 3.1.3. Open Field

**Figure 2.**
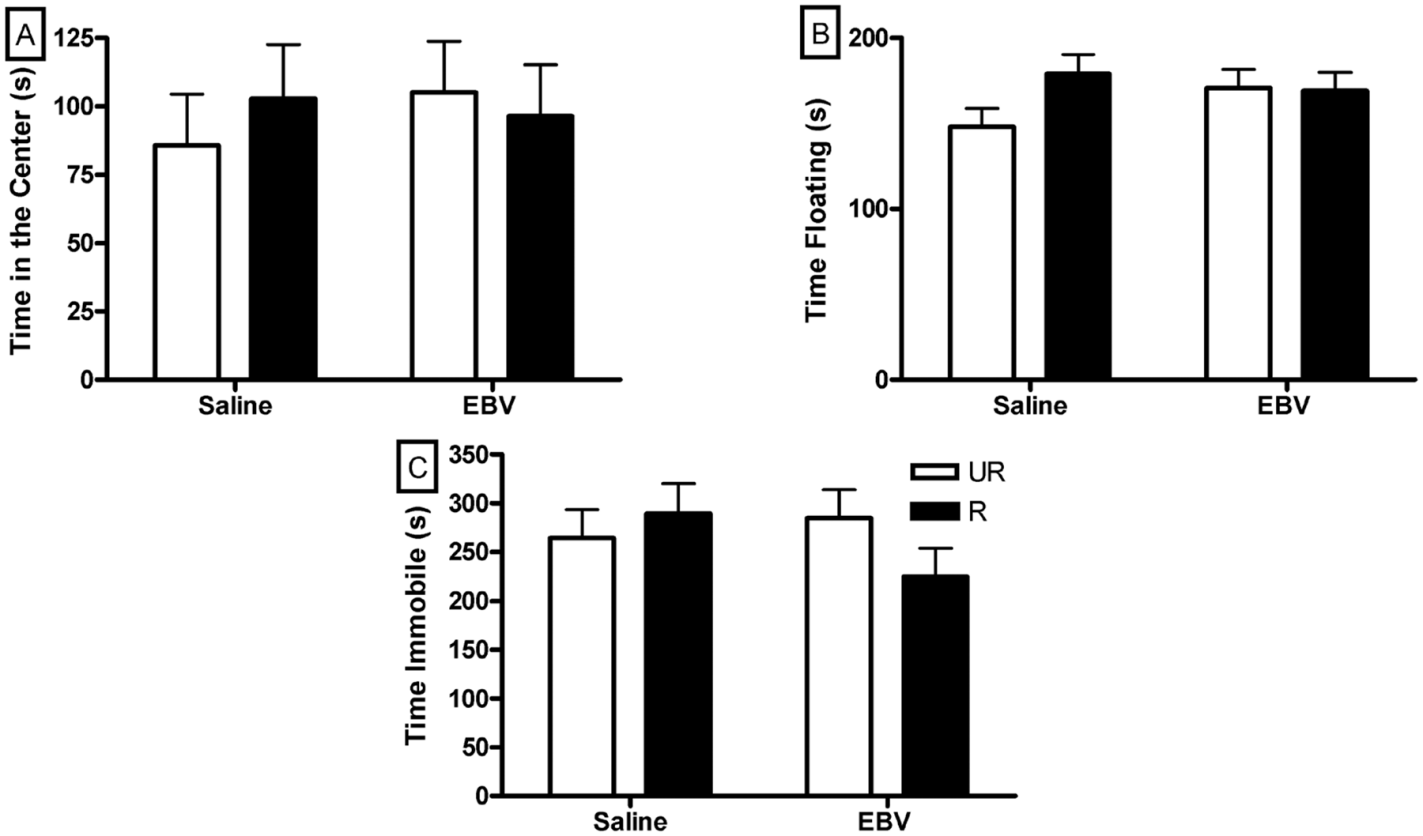
In the one day experiment, neither restraint nor EBVdUTPase injections affected anxiety- or depressive-like behaviors. Time spent in the center of the open field (±SEM) (A); Time spent floating in the forced swim test (±SEM) (B); Time spent immobile during the tail suspension test (±SEM) (C). Restrained (R) and unrestrained (UR) (n = 10/group except the restrained saline group, n = 9).

Percent of time in the center of the open field was similar between saline and EBV-encoded dUTPase injected mice (*p* > 0.05, Figure 2(A)). Restrained mice displayed a similar level of anxiety-like behavior in the open field compared to unrestrained mice, regardless of saline or EBV-encoded dUTPase injection (*p* > 0.05, Figure 2(A)). Neither restraint nor EBV-encoded dUTPase injection altered total movements in the open field (*p* > 0.05, data not shown).

#### 3.1.4. Forced Swim Test

Time floating (Figure 2(B)), climbing or swimming in the forced swim test was similar between saline and EBV-encoded dUTPase injected mice (*p* > 0.05 in each case). Restrained mice and unrestrained mice did not differ in time floating, climbing or swimming (*p* > 0.05 for all comparisons).

#### 3.1.5. Tail Suspension Test

EBV-encoded dUTPase injected mice and saline injected mice spent similar amounts of time immobile during the tail suspension test (*p* > 0.05, Figure 2(C)). Restrained and unrestrained mice spent similar amounts of time immobile during the tail suspension test (*p* > 0.05, Figure 2(C)).

#### 3.1.6. Inflammatory Gene Expression

**Figure 3.**
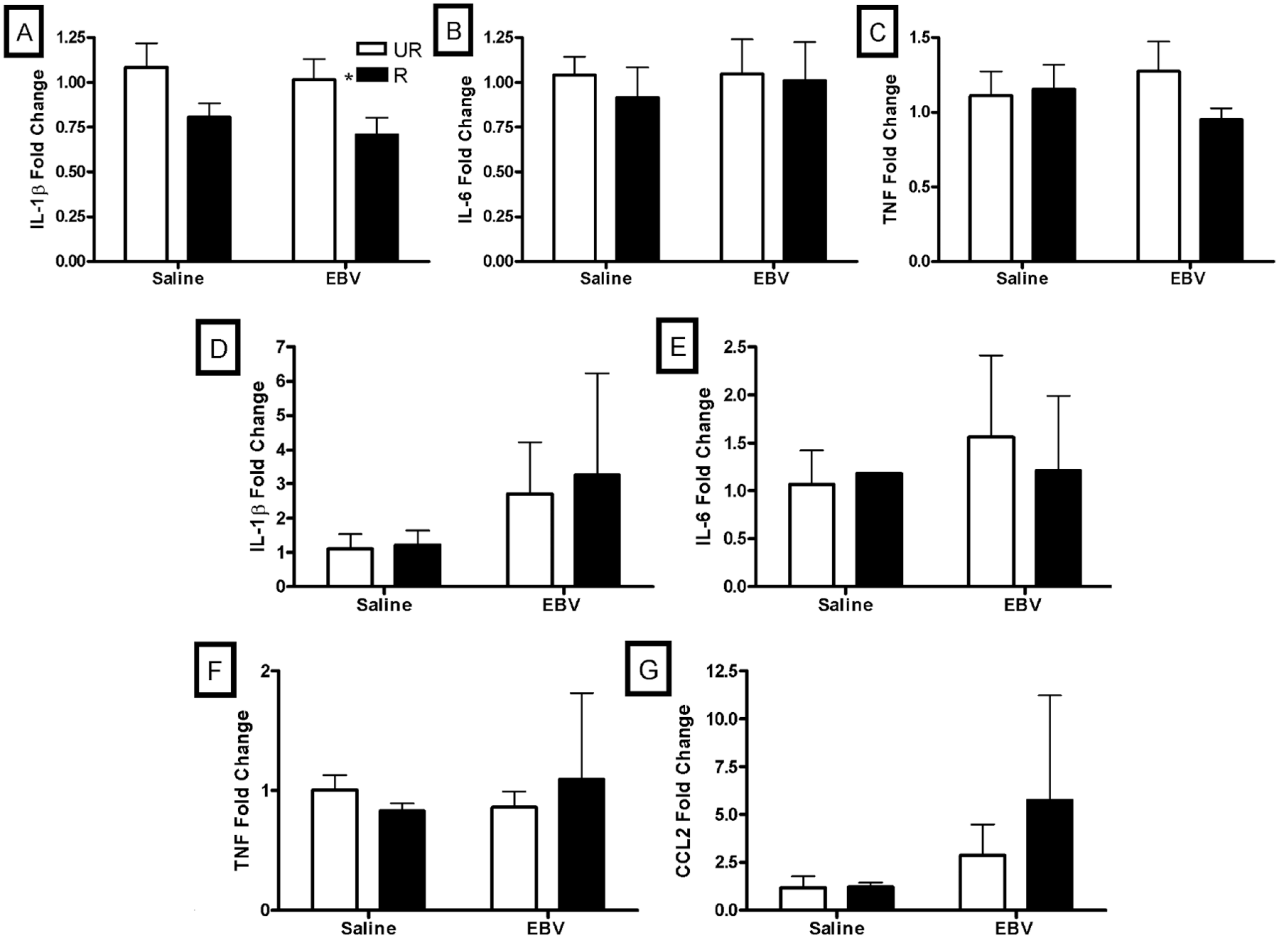
Fold change in inflammatory gene expression in the spleen and inguinal and popiteal pooled lymph nodes in the one-day experiment. Restraint decreased gene expression of interleukin (IL)-1*β* in the spleen (A). Expression of IL-6 (B) and tumor necrosis factor (TNF) (C) were not altered in the spleen with EBVdUTPase injections or restraint (n = 10/group except the restrained saline group, n = 9). Expression of IL-1*β*D), IL-6 (E), TNF (F), and chemokine C-C motif ligand 2 (CCL2) (G) in the pooled inguinal and popliteal lymph nodes was similar between saline and EBVdUTPase injected as well as unrestrained and restrained mice (n = 10/group except the restrained saline group, n = 9). UR = unrestrained, R = restrained. Significant mean differences, *p* < 0.05, indicated with asterisk (*).

Saline and EBV-encoded dUTPase injected mice had similar IL-1*β* expression in the spleen (*p* > 0.05; Figure 3(A)). IL-1*β* expression in the spleen was increased in restrained mice compared to unrestrained mice (F_1,35_ = 7.22, *p* < 0.05; Figure 3(A)). Saline and EBV-encoded dUTPase injected mice had similar IL-6 expression in the spleen (*p* > 0.05; Figure 3(B)). IL-6 expression in the spleen was similar in restrained and unrestrained mice (*p* > 0.05; Figure 3(B)). Saline and EBV-encoded dUTPase injected mice had similar TNF expression in the spleen (*p* > 0.05; Figure 3(C)). TNF expression in the spleen was similar in restrained and unrestrained mice (*p* > 0.05; Figure 3(C)).

Saline and EBV-encoded dUTPase injected mice had similar IL-1*β* expression in the lymph nodes (*p* > 0.05; Figure 3(D)). IL-1*β* expression in the lymph nodes was similar in restrained and unrestrained mice (*p* > 0.05; Figure 3(D)). Saline and EBV-encoded dUTPase injected mice had similar IL-6 expression in the lymph nodes (*p* > 0.05; Figure 3(E)). IL-6 expression in the lymph nodes was similar in restrained and unrestrained mice (*p* > 0.05; Figure 3(E)). Saline and EBV-encoded dUTPase injected mice had similar TNF expression in the lymph nodes (*p* > 0.05; Figure 3(F)). TNF expression in the lymph nodes was similar in restrained and unrestrained mice (*p* > 0.05; Figure 3(F)). Saline and EBV-encoded dUTPase injected mice had similar CCL2 expression in the lymph nodes (*p* > 0.05; Figure 3(G)). CCL2 expression in the lymph nodes was similar in restrained and unrestrained mice (*p* > 0.05; Figure 3(G)).

### 3.2. Three Days of Treatment

#### 3.2.1. Body Temperature

**Figure 4.**
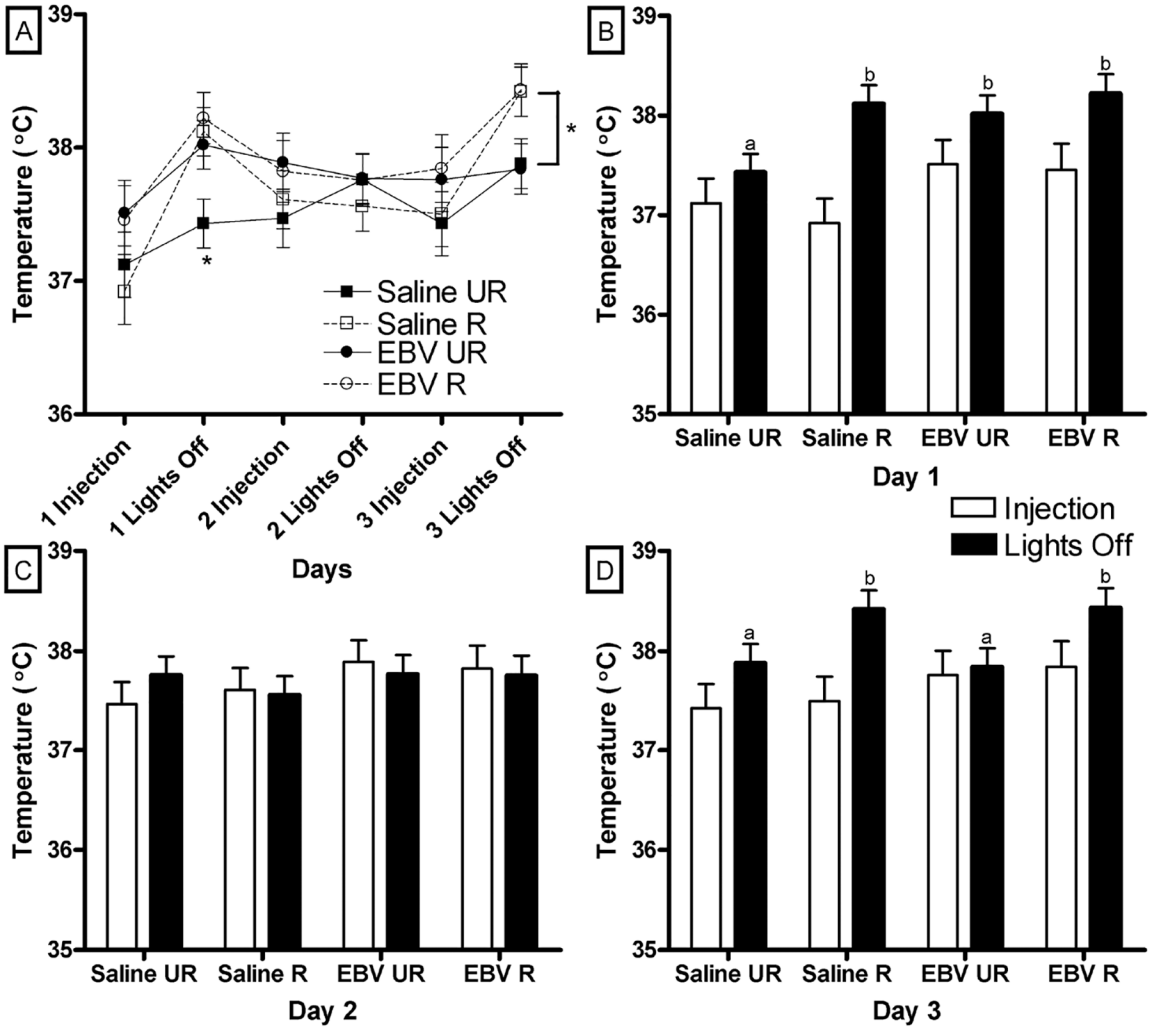
In the three-day experiment, restraint increased temperature right before lights off on days 1 and 3. Body temperature (±SEM °C) across all three days after injections (8:30) and just before lights out (17:30) (A). Body Temperature (±SEM °C) on day 1 (B), day 2 (C) and day 3 of injections (D). Significant mean differences *p* < 0.05 indicated by asterisk (*). Significant mean differences at the lights off time point of *p* < 0.05 indicated by different letters (e.g., a vs. b). Restrained (R) and unrestrained (UR) (n = 10/group).

Temperature was similar between saline and EBV-encoded dUTPase injected mice at both time points on days (8:30 and 17:00 h) 1, 2, and 3 (*p* > 0.05, Figures 4(A)–(D)). Restrained mice had a similar temperature to unrestrained mice on the second day of injections at both time points (Figure 4(C)) and immediately following injections on day 1 and 3 of injections (*p* > 0.05). Restraint increased temperature right before lights off (17:00 h) on days 1 and 3 (F_1,35_ = 5.79, *p* < 0.05, Figure 4(B); F_1,35_ = 8.91, *p* < 0.05, Figure 4(C)). Saline unrestrained mice had a lower temperature than all other groups just before lights off on the first day of injections (*p* < 0.05). Temperature was increased in saline and EBV-encoded dUTPase injected and restrained mice compared to saline and EBV-encoded dUTPase injected unrestrained mice on the third day of injections (*p* < 0.05). One mouse from the EBV restrained group was an outlier based on z score and was removed from temperature analyses.

#### 3.2.2. Body Mass

**Figure 5.**
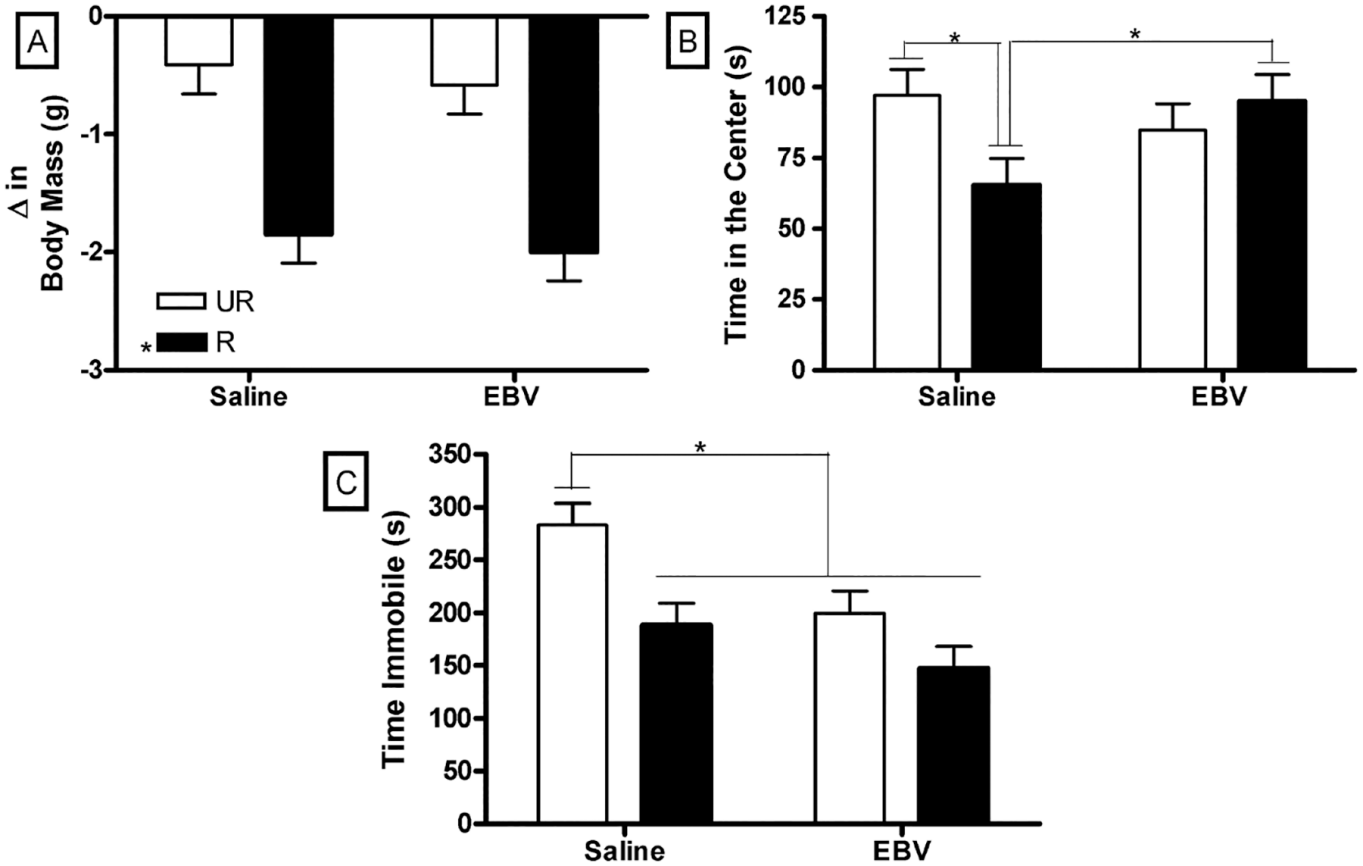
In the three-day experiment, restrained (R) mice lost more weight (±SEM) than unrestrained (UR) mice (A); Restraint decreased time (±SEM) spent in the center of the open field only in saline injected mice (B); Saline injected and unrestrained mice spent more time (±SEM) immobile than all other groups (C). Significant mean differences *p* < 0.05 indicated by asterisk (*) (n = 10/group).

Weight loss did not differ between saline or EBV-encoded dUTPase injected mice (*p* > 0.05). Restrained mice lost more weight than unrestrained mice (F_1,36_ = 34.57, *p* < 0.01, Figure 5(A)). Saline and EBV-encoded dUTPase restrained mice lost more weight than saline and EBV-encoded dUTPase unrestrained mice (*p* < 0.05).

#### 3.2.3. Open Field

Restraint decreased time spent in the center of the open field only in saline injected mice (F_1,36_ = 5.05, *p* < 0.05, Figure 5(B)). Saline injected and restrained mice spent less time in the center of the open field than saline and EBV-encoded dUTPase unrestrained mice (*p* > 0.05). Neither restraint nor EBV-encoded dUTPase injection altered activity in the open field (*p* > 0.05, data not shown).

#### 3.2.4. Tail Suspension Test

EBV-encoded dUTPase injected mice spent less time immobile during the tail suspension test than saline injected mice (F_1,36_ = 9.00, *p* < 0.01, Figure 5(C)). Restrained mice spent less time immobile during the tail suspension test than unrestrained mice (F_1,36_ = 12.69, *p* < 0.01).

#### 3.2.5. Inflammatory Cytokine Gene Expression

**Figure 6.**
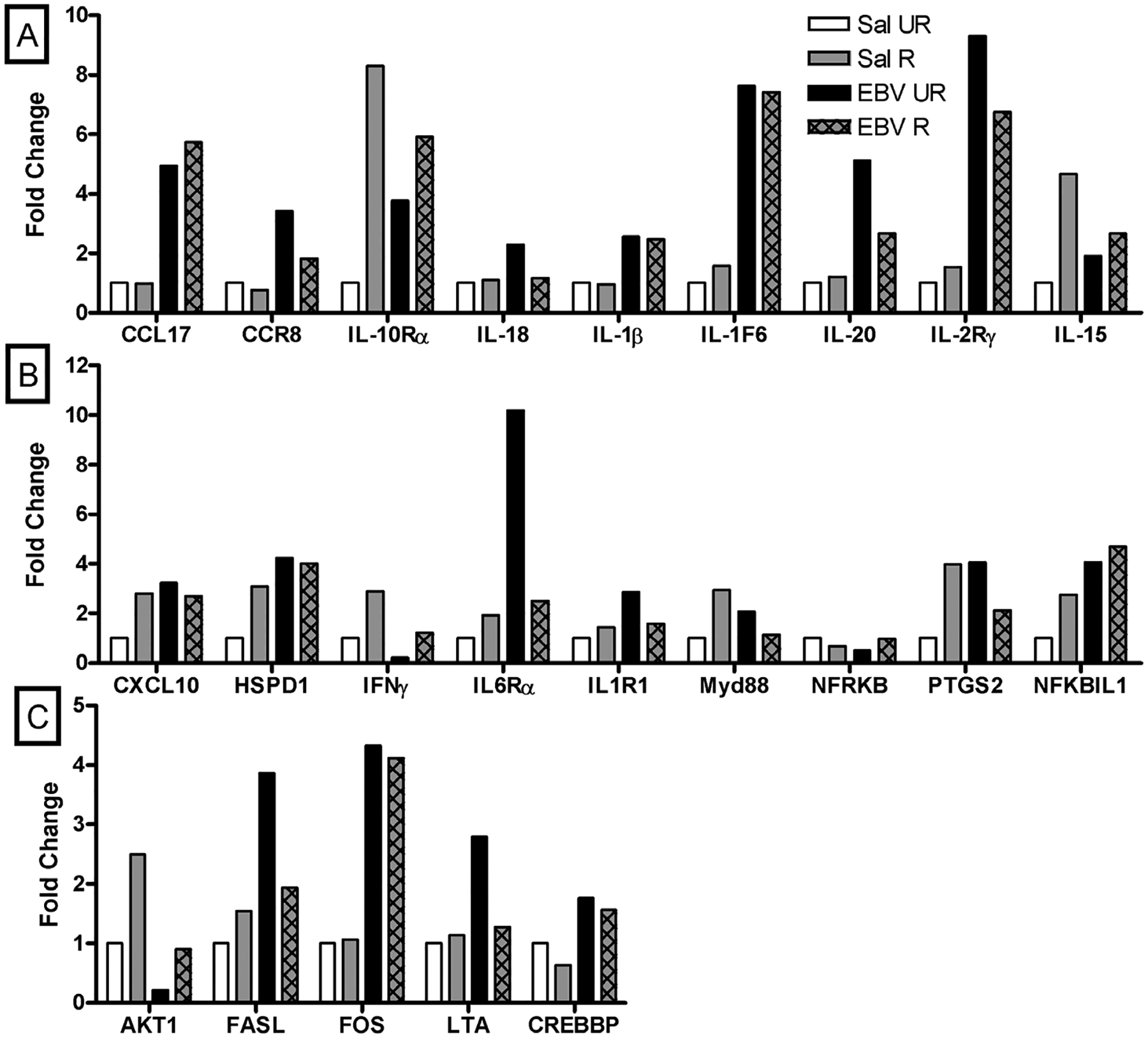
**Fold changes in inflammatory cytokine and receptor expression determined with PCR array in the pooled inguinal and popliteal lymph nodes in the three-day experiment (A).** Fold changes in inflammatory cytokine and receptor expression in saline unrestrained mice compared to EBVdUTPase and unrestrained mice; chemokine (C-C motif) ligand 17 (CCL17), chemokine (C-C motif) receptor 8 (CCR8), interleukin 10 receptor alpha (IL-10R*α*), IL-1*β*, IL-1F6, IL-20 and IL-2R*γ* had a fold change of 2.4 or greater. Fold changes in inflammatory cytokine and receptor expression in saline unrestrained mice compared to EBVdUTPase and restrained mice; CCL17, IL-10R*α*, IL-15, IL-1*β*, IL-1F6, IL-20 and IL-2R*γ* had a fold change of 2.4 or greater. Fold changes in inflammatory cytokine and receptor expression in EBVdUTPase unrestrained mice compared to EBVdUTPase and restrained mice; no genes had a fold change of 2.4 or greater. n = 10/group. **Fold changes in toll-like receptor signaling pathway expression determined with PCR array in the pooled inguinal and popliteal lymph nodes in the three-day experiment (B).** Fold changes in inflammatory cytokine and receptor expression in saline unrestrained mice compared to EBVdUTPase and unrestrained mice; C-X-X motif chemokine ligand 10 (CXCL10), heat shock 60 kDa protein 1 (HSPD1), interleukin 6 receptor alpha (IL-6R*α*, IL-1R1), prostaglandin-endoperoxide synthase 2 (PTGS2) had a fold change of 2.4 or greater. Fold changes in inflammatory cytokine and receptor expression in saline unrestrained mice compared to EBVdUTPase and restrained mice; CXCL10, HSPD1, IL-6R*α*, and NF-kappa-B inhibitor-like protein 1 (NFKBIL1) had a fold change of 2.4 or greater. Fold changes in inflammatory cytokine and receptor expression in saline unrestrained mice compared to saline and restrained mice; HSPD1, interferon gamma (IFN*γ*), myeloid differentiation primary response gene 88 (MyD88), EIF2AK2 had a fold change of 2.4 or greater. Fold changes in inflammatory cytokine and receptor expression in saline restrained mice compared to EBVdUTPase and restrained mice; CXCL10, IFN*γ* NFKBIL1, PTGS2 had a fold change of 2.4 or greater. Fold changes in inflammatory cytokine and receptor expression in EBVdUTPase unrestrained mice compared to EBVdUTPase and restrained mice; IFN*γ* had a fold change of 2.4 or greater. n = 10/group. **Fold changes in nuclear factor kappa B (NFκB) signaling pathway determined with PCR array in the pooled inguinal and popliteal lymph nodes in the three-day experiment (C).** Fold changes in inflammatory cytokine and receptor expression in saline unrestrained mice compared to EBVdUTPase and unrestrained mice; Fas ligand (FASL), FOS, and lymphotoxin a (LTA) had a fold change of 2.4 or greater. Fold changes in inflammatory cytokine and receptor expression in saline unrestrained mice compared to EBVdUTPase and restrained mice; FOS had a fold change of 2.4 or greater. Fold changes in inflammatory cytokine and receptor expression in saline unrestrained mice compared to saline and restrained mice; no genes had a fold change of 2.4 or greater. Fold changes in inflammatory cytokine and receptor expression in saline restrained mice compared to EBVdUTPase and restrained mice; cAMP response element-binding protein (CREBBP) and FOS had a fold change of 2.4 or greater. Fold changes in inflammatory cytokine and receptor expression in EBVdUTPase unrestrained mice compared to EBVdUTPase and restrained mice; protein kinase B 1 (AKT1) had a fold change of 2.4 or greater. n = 10/group.

Fold change in expression of CCL-17, chemokine (C-C motif) receptor (CCR) 8, IL-10R*α*, IL-1*β*, IL-1F6, IL-20, and IL-2R*γ* was greater than 2.4 in EBVdUTPase injected and unrestrained mice than saline injected and unrestrained mice (Figure 6(A)). Fold change in expression of IL-18 was similar in EBVdUTPase injected and unrestrained mice than saline injected and unrestrained mice (Figure 6(A)). Fold change in expression of CCL-17, IL-10R*α*, IL-5, IL-1*β*, IL-1F6, IL-20, and IL-2R*γ* was greater than 2.4 in EBVdUTPase injected and restrained mice than saline injected and unrestrained mice (Figure 6(A)). There were no fold changes greater than 2.4 in expression of IL-13R*α*1, aminoacyl tRNA synthestase complex-interacting multifunctional protein 1 (AIMP1), CCL20, C-X-X motif chemokine ligand 12 (CXCL12), and CXCL13 between EBV unrestrained and EBV restrained mice (Figure 6(A)).

#### 3.2.6. Toll-Like Receptor Expression

Fold change in expression of CXCL10, heat shock 60 kDa protein 1 (HSPD1), IL6R*α*, IL1R1, and prostaglandin-endoperoxide synthase 2 (PTGS2) was greater than 2.4 in EBVdUTPase injected and unrestrained mice than saline injected and unrestrained mice (Figure 6(B)). Fold change in expression of interferon gamma (IFN*γ*, myeloid differentiation primary response gene 88 (MyD88), and (nuclear factor related to kappaB binding protein) NFKRKB was similar in EBVdUTPase injected and unrestrained mice than saline injected and unrestrained mice (Figure 6(B)). Fold change in expression of CXCL10, HSPD1, IL-6R*α*, and NF-kappa-B inhibitor-like protein 1 (NFKBIL1) was greater than 2.4 in EBVdUTPase injected and restrained mice than saline injected and unrestrained mice (Figure 6(B)). Fold change in expression of inhibitor of kappa light polypeptide gene enhancer in B-cells, kinase beta (IKBKB) and PTGS2 was similar in EBVdUTPase injected and restrained mice than saline injected and unrestrained mice (Figure 6(B)). Fold change in expression of HSPD1, IFN*γ*, MyD88, and eukaryotic translation initiation factor 2-alpha kinase 2 (EIF2AK2) was greater than 2.4 in saline injected and restrained mice than saline injected and unrestrained mice (Figure 6(B)). Fold change in expression of CXCL10, IFN*γ*, NFKBIL1, and PTGS2 was greater than 2.4 in EBVdUTPase injected and restrained mice than saline injected and restrained mice (Figure 6(B)). Fold change in expression of MyD88, EIF2AK2 and ubiquitin-conjugating enzyme E2 variant 1 (UBE2V1) was similar in EBVdUTPase injected and restrained mice than saline injected and restrained mice (Figure 6(B)). Fold change in expression of IFN*γ* was increased more than 2.4 in EBVdUTPase and restrained mice than EBVdUTPase and unrestrained mice (Figure 6(B)). Fold change in expression of conserved helix-loop-helix ubiquitous kinase (CHUK) and IL-6R*α* was similar in EBVdUTPase and restrained mice than EBVdUTPase and unrestrained mice (Figure 6(B)).

#### 3.2.7. Nuclear Factor Kappa B Expression

Fold change in expression of Fas ligand (FASL), FOS, and lymphotoxin a (LTA) was greater than 2.4 in EBVdUTPase injected and unrestrained mice than saline injected and unrestrained mice (Figure 6(C)). Fold change in expression of protein kinase B 1 (AKT1) was similar in EBVdUTPase injected and unrestrained mice than saline injected and unrestrained mice (Figure 6(C)). Fold change in expression of FOS was greater than 2.4 in EBVdUTPase injected and restrained mice than saline injected and unrestrained mice (Figure 6(C)). Fold change in expression of IL1R1 and IRF1 was similar in EBVdUTPase injected and restrained mice than saline injected and unrestrained mice (Figure 6(C)). Fold change in expression of AKT1 and interferon regulatory factor 1 (IRF1) was similar in saline injected and restrained mice and saline injected and unrestrained mice (Figure 6(C)). Fold change in expression of cAMP response element-binding protein (CREBBP) and FOS was greater than 2.4 in EBVdUTPase injected and restrained mice than saline injected and restrained mice (Figure 6(C)). Fold change in expression of AKT1 and caspase 8 (CASP8) was similar in EBVdUTPase injected and restrained mice than saline injected and restrained mice (Figure 6(C)). Fold change in expression of AKT1 was increased more than 2.4 in EBVdUTPase and restrained mice than EBVdUTPase and unrestrained mice (Figure 6(C)). Fold change in expression of LTA was similar in EBVdUTPase and restrained mice than EBVdUTPase and unrestrained mice (Figure 6(C)).

## 4. Discussion

Most humans have experienced infection with EBV and the symptoms associated with CFS can be exacerbated by stress [40]. EBV-encoded dUTPase can elicit a sickness response independent of viral replication [15]. To assess changes in anxiety-like behavior and sickness responses, mice were injected with EBV-encoded dUTPase for one or three days and restrained 3 h each day. We hypothesized that restraint would exacerbate sickness behavior elicited by EBV-encoded dUTPase. There were no behavioral differences in the one day experiment indicating that restraint stress had no effect when experienced acutely but produced a sickness response after repeated exposure.

Previously, 20 μg EBV-encoded dUTPase injections induced a sickness response in mice; temperature was measured twice daily, but EBV-encoded dUTPase-induced increases in body temperature did not occur until the fifth day and significant temperature differences were intermittent through the remainder of the 14 days of injections [15]. Additionally, decreased locomotor activity following EBV-encoded dUTPase injections did not occur until the second week of injections [15]. Similarly, in the current study one or three days of injections did not reduce locomotor activity or increase body temperature in EBV-encoded dUTPase injected mice when temperature was assessed twice daily. However, three days of restraint increased body temperature during the transition to darkness. The normal diurnal variation in body temperature was observed on all days, with higher body temperatures at the onset of the dark phase than during the light phase [41]. One and three days of restraint decreased body mass gain, however, food intake was not measured in this study; restraint stress is associated with reduced food intake and can be interpreted as a sickness response [18]. These data suggest that extended injections of EBV-encoded dUTPase are required to increase body temperature and reduce locomotor activity.

Even though it required more than five days for any sickness behavior to occur in the previous study, immune tissues, namely spleen and lymph nodes, had reduced response to challenge with the mitogen concanavalin-A (Con-A) and LPS after three days of 10 μg EBV-encoded dUTPase injections [15]. Synthesis of interferongamma was reduced in the supernatant from spleen and lymph nodes treated with Con-A and LPS in EBV-encoded dUTPase injected mice one and three days after the final injection [15]. Similarly, three days of 10 μg EBV-encoded dUTPase injections compared to saline injections upregulated expression of some inflammatory cytokines and receptors, toll-like receptor signaling pathway elements, and NFκB signaling pathway elements in the pooled inguinal and popliteal lymph nodes. Injections of 10 μg EBV-encoded dUTPase also provoked sickness responses in a previous study using chronic restraint stress [18]. Spleen and pooled lymph nodes after one day of EBVdUTPase injections did not alter cytokine expression; however, one day of restraint stress reduced IL-1*β* gene expression in the spleen. IL-1*β* mRNA is reduced by one day of restraint; however, five days of restraint increased IL-1*β* mRNA expression following a cutaneous wound [42]. The above data combined with our current behavioral data suggest that EBV-encoded dUTPase induced cellular immune changes occur before the manifestation of behavioral changes, whereas restraint stress appears to induce behavioral changes when experienced repeatedly.

Glucocorticoids are associated with immunosuppression, but glucocorticoids can also induce a cytokine response [7]. Prolonged exposure to the open field acts as a stressor in rats, leading to increased plasma IL-6 and body temperature [9]. Independent of an immune challenge, chronic unpredictable stress in rats induces cognitive-deficits and anxiety-like behavior that can be prevented with chronic antidepressant treatment [43]. The addition of an immune challenge, LPS, following chronic unpredictable stress increased the sickness response, including lethargy relative to non-stressed animals [44]. Similarly, independent of immune challenge, exposure to an inescapable tail-shock increases core body temperature and decreases activity; if rats are then subjected to LPS challenge an increased sickness response is observed [45]. These anxiety-like behavior and cognitive deficits caused by repeated stress exposure are similar to sickness behaviors elicited by LPS [46] [47]. These previous studies support our current results that indicate repeated exposure to restraint stress induces a sickness response.

Both restraint stress and sickness can alter anxiety-like behavior [4] [46]. As expected, restraint stress increased anxiety-like behavior, but in the current study was driven by saline injected mice. Contrary to expectation, EBV-encoded dUTPase injected mice that were restrained did not differ from unrestrained mice regardless of injection. Again these data support the notion that manifestation of behavioral changes requires a longer duration of EBV-encoded dUTPase injection.

## 5. Conclusion

In conclusion, three, but not one, days of restraint stress elicited a sickness response regardless of whether mice were injected with saline or EBV-encoded dUTPase. Previous data have implicated a role for this viral protein in the induction of sickness behavior in mice; however, behavioral differences were not observed following EBV-encoded dUTPase injection in the current study [15]. Likely more than three consecutive injections of EBVdUTPase are required to elicit a sickness response, whereas, three days of repeated restraint stress can elicit a sickness response.
